# Mortality Burden for Patients With Untreated Aortic Regurgitation

**DOI:** 10.1016/j.jacadv.2024.101228

**Published:** 2024-09-06

**Authors:** Philippe Généreux, Nicholas S. Amoroso, Vinod H. Thourani, Evelio Rodriguez, Rahul P. Sharma, Duane S. Pinto, Michelle Kwon, Michael Dobbles, Patricia A. Pellikka, Linda D. Gillam

**Affiliations:** a Department of Cardiovascular Medicine, Morristown Medical Center, Morristown, New Jersey, USA; b Division of Cardiology, Department of Medicine, Medical University of South Carolina, Charleston, South Carolina, USA; c Department of Cardiovascular Surgery, Marcus Valve Center, Piedmont Heart Institute, Atlanta, Georgia, USA; d Ascension Saint Thomas Hospital, Cardiac Surgery, Nashville, Tennessee, USA; e Division of Cardiovascular Medicine, Stanford University School of Medicine, Stanford, California, USA; f Division of Cardiovascular Medicine, Beth Israel Deaconess Medical Center and Harvard Medical School, Boston, Massachusetts, USA; g JenaValve Technology, Inc, Irvine, California, USA; h egnite Inc, Aliso Viejo, California, USA; i Department of Cardiovascular Medicine, Mayo Clinic, Rochester, Minnesota, USA

**Keywords:** aortic insufficiency, aortic regurgitation, aortic valve, aortic valve replacement, database, natural language processing

## Abstract

**Background:**

Aortic valve replacement (AVR) is indicated in patients with severe aortic regurgitation (AR); however, certain clinical factors may identify patients with less-than-severe AR at high mortality risk if untreated.

**Objectives:**

The authors sought to characterize key associations with mortality across the spectrum of AR in patients not treated with AVR from a large, contemporary database.

**Methods:**

We analyzed patients >18 years of age with documented AR assessment in a deidentified real-world data set from 27 U.S. institutions with appropriate permissions (egnite Database, egnite, Inc). Diagnosed AR severity was extracted from echocardiographic reports using a natural language processing–based algorithm. Cox multivariable analysis modeled the impact of key factors on untreated mortality according to AR severity.

**Results:**

In total, 81,378 patients were included for analysis. Hazard ratios for mortality were 1.26 (95% CI: 1.18-1.35) and 2.37 (95% CI: 1.96-2.87) for moderate and severe AR, respectively. Other significant associations included left ventricular (LV) ejection fraction ≤55% (1.09 [95% CI: 1.02-1.15]), LV dilation (1.34 [95% CI: 1.21-1.48]), left atrial dilation (1.09 [95% CI: 1.03-1.16]), atrial fibrillation (1.11 [1.04-1.17]), and elevated B-type natriuretic peptide/N-terminal pro-B-type natriuretic peptide (1.71 [95% CI: 1.60-1.84]). Modeled mortality risk increased with the presence of these key factors both alone and in combination.

**Conclusions:**

In patients with untreated AR, LV remodeling, left atrial remodeling, and other markers of cardiac damage are associated with substantial mortality risk, both for severe and moderate AR. Further study is needed to determine whether AVR is warranted in patients with less-than-severe AR with at-risk factors.

Aortic regurgitation (AR) is a frequent valve disease, with up to 4.5% of patients older than 65 years having moderate or severe AR.^1^^,^^2^ AR is associated with left ventricle (LV) volume overload and, if left untreated, leads to LV enlargement, pulmonary congestion, decreased cardiac output, and decreased LV ejection fraction (LVEF). Retrospective data from small series have demonstrated increased mortality with chronic severe AR, especially if associated with symptoms,^3^ LV dilation,^4^, ^5^, ^6^, ^7^, ^8^ and reduced LVEF.^9^, ^10^, ^11^

The American College of Cardiology/American Heart Association guidelines recommend aortic valve replacement (AVR) as a Class 1 recommendation for patients with severe AR associated with either symptoms or LVEF £55%, as a Class 2a recommendation for patients with systolic LV dilation (LV end-systolic diameter >50 mm or indexed LV end-systolic diameter >25 mm/m^2^), and as a class 2b recommendation when there is a progressive decline in LVEF on at least 3 serial studies to the low–normal range (LVEF 55%-60%) or a progressive increase in LV dilation into the severe range (LV end-diastolic dimension >65 mm).^12^ AVR for moderate AR could be considered (class 2a) in the context where other open-heart surgery is needed, such as coronary artery bypass, mitral valve replacement, or root enlargement. Clinically, it is often challenging to determine the severity of AR, and especially to distinguish between moderate and severe AR. Similarly, patients with moderate AR may not tolerate the associated increase in LV volume and may become symptomatic or have LV enlargement while still being considered nonsevere. In the present study, we aimed to assess the rates of mortality across the spectrum of untreated AR severity and to identify the determinants of mortality from a contemporary, large, real-world database.

## Methods

At the time of this study, a total of 1,223,017 patients >18 years of age with a qualifying documented diagnosis of AR (whether none, mild, mild-to-moderate, moderate, moderate-to-severe, or severe) per echocardiographic reports were represented in the egnite database (egnite) from 27 institutions with appropriate permissions. Exemption from Institutional Review Board review for this study was obtained from the WIRB-Copernicus Group, and all deidentified data sets used were compliant with the Health Insurance Portability and Accountability Act (HIPAA). Data were prepared for the present study following initial data quality assessments by a clinical team and evaluated for study inclusion and exclusion criteria.

The key inclusion criterion for this study was at least one instance of documented assessment for AR as extracted from the text of echocardiographic reports using a natural language processing (NLP)–based algorithm, with severity specified if a diagnosis of AR was present. The NLP-based algorithm has been clinically reviewed and validated, as previously reported.^13^ All available echocardiogram types documented for a given patient were used to identify AR diagnosis, and in the event of more than one available diagnosis, the date of the first report with a documented assessment of AR of any severity was counted as the study index date. Eligible index dates ranged from January 1, 2018 to October 5, 2023 in the study data set lock (but all true dates were eliminated at data set extraction to help ensure irreversible deidentification). Key exclusion criteria comprised a documented diagnosis of moderate or greater aortic stenosis (given that such patients would be expected to exhibit substantial calcification of the aortic valve, ie, not representative of the population of interest in the present study, which is focused on noncalcific AR), metastatic cancer, and/or Alzheimer’s or other dementia (which may impact a therapeutic decision). Comorbidities, documented treatment events, and other key patient characteristics were identified according to relevant International Classification of Diseases-10th Revision/Current Procedural Terminology codes and/or echocardiographic report data, with all definitions adjudicated by a clinical reviewer as well as a medical coding expert where relevant. Information on patient death was extracted from medical records (ie, as documented by each site).

To generate a fully time-varying data set required for this study within given computing constraints, statistically random down-sampling of the population was performed to no more than 20,000 patients per cohort when stratified by greatest AR severity. The final data set not only treated diagnosis of AR as time-varying (eg, if a patient’s first documented diagnosis was no/mild AR and a subsequent echocardiographic study yielded a diagnosis of moderate AR, this was captured accordingly in the data set—this patient would contribute data to the no/mild AR cohort until they were diagnosed with moderate AR, at which point in time the patient would cease to contribute data to the no/mild AR cohort and instead contribute to the moderate AR cohort), but it also treated all patients’ comorbidities and other prespecified characteristics of interest as time-varying as well.

### Statistical analysis

Patient characteristics were reported as n (%) for categorical variables and as mean ± SD or median (IQR) for continuous variables, as appropriate. Cox multivariable analysis was used to model the impact of key factors of interest on all-cause untreated mortality, where patients were censored at the time of treatment with AVR or at last documented clinical encounter. Prespecified factors of interest included LV dilation (LV end-systolic dimension index >25 mm/m^2^ and/or LV end-systolic volume index ≥45 mL/m^2^]),^14^ LVEF ≤55%, left atrial (LA) dilation (LA volume index >34 mL/m^2^), atrial fibrillation, and elevated B-type natriuretic peptide (BNP) (≥400 pg/mL), and/or N-terminal pro-B-type natriuretic peptide level (NT-proBNP) (≥1,500 pg/mL).

Hazard ratios associated with key prespecified covariates of interest, after adjustment for other potential confounders such as age, patient sex, and other comorbidities or available echocardiographic characteristics of potential relevance, were then used to estimate the modeled survival functions and, inversely, the modeled mortality functions for a patient with or without key factors or combinations of factors. All analyses were performed using Databricks Runtime, version 13.4 LTS (Apache Spark 3.4.1, Scala 2.12), R, version 4.2.2, survival package, version 3.5.3.

## Results

### Study population

A total of 81,378 patients met all eligibility criteria, including documented AR severity, and were included in the population for analysis after down-sampling (Supplemental Figure 1). Median time to patient treatment with AVR, last documented clinical encounter, or death was 18.4 (IQR: 6.0-34.1) months. Table 1 shows baseline characteristics per AR severity as of study entry. In general, patients with more severe AR were younger, were more frequently male, had lower documented prevalence of coronary artery disease or atrial fibrillation, and more often exhibited reduced LVEF, increased BNP and/or NT-proBNP, and recent hospitalization with heart failure.

**Table 1:** Baseline Characteristics of the Study Population^a^ per Aortic Regurgitation Severity

Characteristics^a^	No/Mild AR (n = 47,644)	Mild-to-Moderate AR (n = 17,640)	Moderate AR (n = 13,316)	Moderate-to-Severe AR (n = 1,741)	Severe AR (n = 1,037)	*P* Value
Age, y	67.3 ± 15.0	74.0 ± 12.3	73.7 ± 12.9	69.4 ± 14.8	62.4 ± 16.5	<0.001
Sex^b^						<0.001
Female	24,122 (50.6)	9,139 (51.8)	7,087 (53.2)	761 (43.7)	344 (33.2)	
Male	23,513 (49.4)	8,498 (48.2)	6,227 (46.8)	980 (56.3)	693 (66.8)	
Atrial fibrillation	9,958 (20.9)	4,526 (25.7)	3,303 (24.8)	378 (21.7)	151 (14.6)	<0.001
Prior stroke	4,191 (8.8)	1,705 (9.7)	1,259 (9.5)	143 (8.2)	59 (5.7)	<0.001
COPD	4,641 (9.7)	1,750 (9.9)	1,473 (11.1)	177 (10.2)	66 (6.4)	<0.001
COPD on O_2_	589 (1.2)	184 (1.0)	145 (1.1)	9 (0.5)	7 (0.7)	0.009
CAD	13,875 (29.1)	5,602 (31.8)	4,120 (30.9)	474 (27.2)	233 (22.5)	<0.001
Prior MI	2,965 (6.2)	1,113 (6.3)	789 (5.9)	89 (5.1)	42 (4.1)	0.009
Prior PCI	1,312 (2.8)	439 (2.5)	300 (2.3)	35 (2.0)	9 (0.9)	<0.001
Prior CABG	345 (0.7)	103 (0.6)	71 (0.5)	8 (0.5)	3 (0.3)	0.025
New-onset MI	21 (<0.1)	4 (<0.1)	2 (<0.1)	0 (0.0)	0 (0.0)	0.338
Diabetes	10,624 (22.3)	2,855 (16.2)	1,976 (14.8)	227 (13.0)	102 (9.8)	<0.001
Chronic kidney disease	6,683 (14.0)	2,799 (15.9)	2,166 (16.3)	265 (15.2)	146 (14.1)	<0.001
HF-related hospitalization in prior year	4,713 (9.9)	1,885 (10.7)	1,675 (12.6)	247 (14.2)	209 (20.2)	<0.001
Liver disease	2,706 (5.7)	755 (4.3)	555 (4.2)	88 (5.1)	45 (4.3)	<0.001
Moderate or greater MR	168 (0.4)	32 (0.2)	35 (0.3)	1 (0.1)	4 (0.4)	0.002
Moderate or greater TR	157 (0.3)	26 (0.1)	20 (0.2)	3 (0.2)	4 (0.4)	<0.001
LVEF ≤55%	897 (1.9)	167 (0.9)	137 (1.0)	12 (0.7)	15 (1.4)	<0.001
LV dilation (LVESDi >25 mm/m^2^ and/or LVESVi ≥45 mL/m^2^)	38 (0.1)	9 (0.1)	8 (0.1)	0 (0.0)	2 (0.2)	0.268
LA dilation (LAVi >34 mL/m^2^)	313 (0.7)	44 (0.2)	39 (0.3)	5 (0.3)	7 (0.7)	<0.001
Severe pulmonary arterial hypertension (PASP ≥60 mm Hg)	7 (<0.1)	0 (0.0)	0 (0.0)	0 (0.0)	1 (0.1)	0.015
Moderate or greater RV dysfunction (per echocardiographic report)	58 (0.1)	6 (<0.1)	2 (<0.1)	1 (0.1)	0 (0.0)	<0.001
TAPSE <1.6 cm	96 (0.2)	16 (0.1)	2 (<0.1)	0 (0.0)	0 (0.0)	<0.001
Elevated BNP/NT-proBNP (BNP ≥400 pg/mL and/or NT-proBNP ≥1,500 pg/mL)	3,942 (8.3)	1,839 (10.4)	1,547 (11.6)	230 (13.2)	143 (13.8)	<0.001

Values are mean ± SD or n (%).

AR = aortic regurgitation; BNP = B-type natriuretic peptide; CABG = coronary artery bypass graft; CAD = coronary artery disease; COPD = chronic obstructive pulmonary disease; HF = heart failure; LA = left atrial; LAVi = left atrial volume index; LV = left ventricular; LVEF = left ventricular ejection fraction; LVESDi = left ventricular end-systolic dimension index; LVESVi = left ventricular end-systolic volume index; MI = myocardial infarction; MR = mitral regurgitation; NT-proBNP = N-terminal pro-B-type natriuretic peptide; PASP = pulmonary arterial systolic pressure; PCI = percutaneous coronary intervention; RV = right ventricle; TAPSE = tricuspid annular plane systolic excursion; TR = tricuspid regurgitation.

a Characteristics of modeled study population as of their index (ie, first) echocardiographic report (note: per study design, patients without calcific aortic valve disease, metastatic cancer, or Alzheimer’s/dementia).

b Across cohorts, 9 (<0.1%), 3 (<0.1%), and 2 (<0.1%) patients with none/mild, mild-to-moderate, or moderate AR, respectively, were other/unknown.

### Mortality for patients with untreated AR

Estimated all-cause untreated mortality associated with AR diagnosis of none/mild, mild-to-moderate, moderate, moderate-to-severe, or severe was 7.1% (95% CI: 6.8%-7.3%), 9.5% (95% CI: 9.0%-10.0%), 10.9% (10.2%-11.5%), 10.9% (95% CI: 8.9%-12.7%), and 15.4% (95% CI: 12.5%-18.2%), respectively, at 2 years postindex (Table 2, Supplemental Figure 2).

**Table 2:** 2-Year Observed Mortality Rates for the Study Population^a^, Stratified by Aortic Regurgitation Severity

AR Severity	2-Year Mortality Rate (95% CI)
None/mild	7.1% (6.8%-7.3%)
Mild-to-moderate	9.5% (9.0%-10.0%)
Moderate	10.9% (10.2%-11.5%)
Moderate-to-severe	10.9% (8.9%-12.7%)
Severe	15.4% (12.5%-18.2%)

AVR = aortic valve replacement; other abbreviation as in Table 1.

a Mortality without AVR for modeled study population (note: per study design, patients with predominantly non-calcific aortic valve disease, metastatic cancer, or Alzheimer’s/dementia) per Kaplan-Meier estimates with first documented AR diagnosis as index date and AR severity treated as a time-varying covariate.

### Modeling analysis of key factors

**Figure 1:**
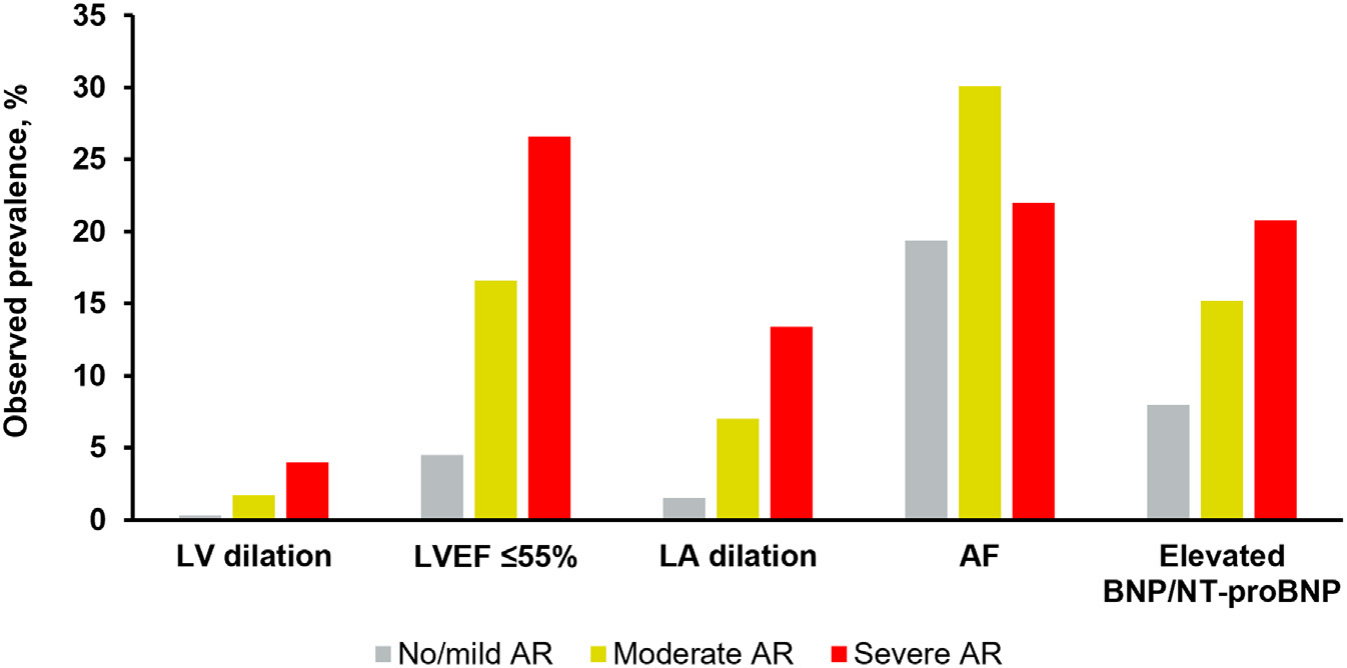
**Proportion of Patients Exhibiting Individual Key Factors∗ at Time of Worst AR Diagnosis Within Study Window** ∗Key factors defined as follows: LV dilation, as LVESDi >25 mm/m^2^ and/or LVESVi ≥45 mL/m^2^; LA dilation, as LAVi >34 mL/m^2^; AF, per International Classification of Diseases-10th revision codes; elevated BNP/NT-proBNP, as BNP ≥400 pg/mL and/or NT-proBNP ≥1,500 pg/mL. AF = atrial fibrillation; AR = aortic regurgitation; BNP = B-type natriuretic peptide; LA = left atrial; LAVi = left atrial volume index; LV = left ventricular; LVEF = left ventricular ejection fraction; LVESDi = left ventricular end-systolic dimension index; LVESVi = left ventricular end-systolic volume index; NT-proBNP = N-terminal pro-B-type natriuretic peptide.

**Figure 2:**
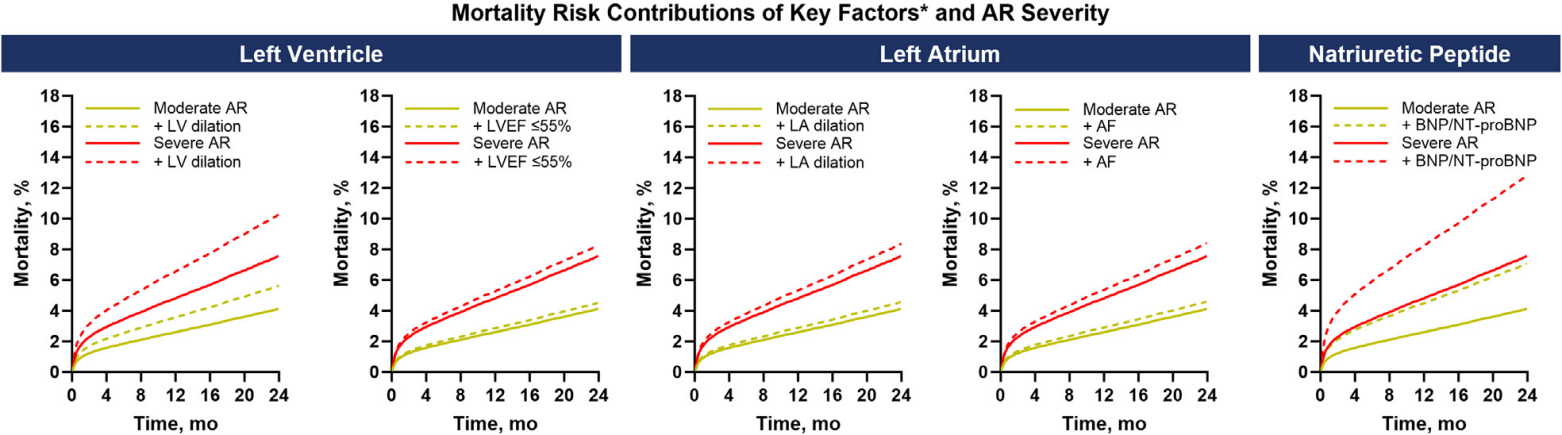
**Modeled Mortality Risk Contributions of Key Factors∗ and AR Severity** ∗Key factors defined as follows: LV dilation, as LVESDi >25 mm/m^2^ and/or LVESVi ≥45 mL/m^2^; LA dilation, as LAVi >34 mL/m^2^; AF, per International Classification of Diseases-10th revision codes; BNP/NT-proBNP, as elevated BNP ≥400 pg/mL and/or NT-proBNP ≥1,500 pg/mL. Abbreviations as in Figure 1.

All degrees of AR severity were associated with increased relative risk of mortality. After adjustment for patient age, sex, key comorbidities, and other echocardiographic characteristics, each of the prespecified factors of interest was also associated with increased mortality risk, with associated HRs of 1.09 (95% CI: 1.02-1.15) for LVEF ≤55%, 1.34 (95% CI: 1.21-1.48) for LV dilation, 1.09 (95% CI: 1.03-1.16) for LA dilation, 1.11 (95% CI: 1.04-1.17) for atrial fibrillation, and 1.71 (95% CI: 1.60-1.84) for elevated BNP/NT-ProBNP (Central Illustration, Supplemental Table 1). The prevalence of these key factors tended to be greater with greater AR severity and over time, and particularly in patients with moderate or greater AR when analyzed cross-sectionally at each patient’s worst documented AR severity in their time series (Figure 1). Modeled mortality risk increased for both patients with moderate AR and severe AR with the presence of these key factors (Figure 2), either alone or in combination, such that several scenarios for patients with moderate AR demonstrated a comparable or greater mortality risk to modeled patients with severe AR (Central Illustration).

**Central Illustration:**
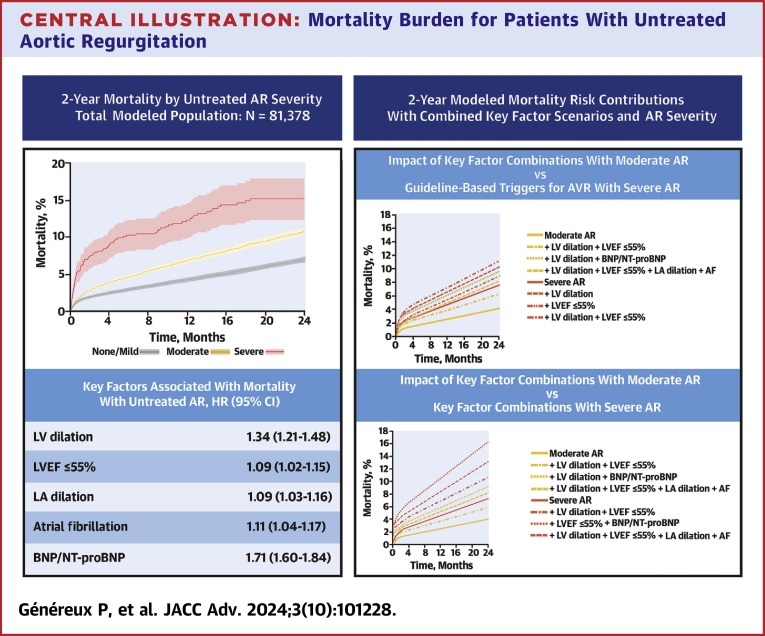
**Mortality Burden for Patients With Untreated Aortic Regurgitation** Impact of untreated aortic regurgitation according to severity and factors associated with 2-year mortality. (Top left) Kaplan-Meier summary analysis of observed mortality with untreated aortic regurgitation over up to 2 years of follow-up after the index documented diagnosis for cohorts of interest, with censoring at last documented clinical encounter or treatment with aortic valve replacement. Analysis of all possible documented severities is also available as Supplemental Material. Because of the time-varying nature of the data set for this study, in this analysis, Aortic regurgitation severity is treated as a time-varying covariate. (Bottom left) Modeled hazards for key factors of interest in this study. (Right) 2-year modeled mortality according to different AR severities and key factors of interest. key factors defined as follows: LV dilation, as LVESDi >25 mm/m^2^ and/or LVESVi ≥45 mL/m^2^; LA dilation, as LAVi >34 mL/m^2^; AF, per International Classification of Diseases-10th revision codes; BNP/NT-proBNP, as elevated BNP ≥400 pg/mL and/or NT-proBNP ≥1,500 pg/mL. Abbreviations as in Figure 1.

## Discussion

To the best of our knowledge, the current study represents, by far, the largest cohort of patients with a diagnosis of AR to be presented. From a contemporary database using NLP to extract diagnosed AR severity, we demonstrated that mortality increases across the spectrum of AR severity and that certain factors, beyond the factors proposed in guidelines,^12^ increase mortality risk among patients with untreated moderate and severe AR.

While the American College of Cardiology/American Heart Association guidelines recommend AVR for patients with severe AR only, our study highlights factors that are associated with increased mortality risk, even when AR is less than severe (moderate). Indeed, the presence of significant LV dilation or LVEF ≤55% was associated with poor prognosis, suggesting potentially compounded risk for patients with untreated moderate to severe AR with these clinical characteristics. Other factors, such as atrial fibrillation and elevated cardiac biomarkers (BNP/NT-proBNP), that are not identified as classic “triggers” for AVR were shown to increase mortality risk for this patient population. Atrial fibrillation is a well-known trigger for intervention among patients with severe mitral regurgitation but not for significant AR or aortic stenosis. Similarly, elevated BNP represents a class 2b indication for AVR among patients with severe aortic stenosis and no symptoms but not for AR. Our findings highlight guideline gaps regarding patient risk stratification and, potentially, treatment recommendation for patients presenting with moderate or severe AR. Prospective studies are needed to confirm these findings.

The presence of LA “disease” (dilation and atrial fibrillation), LV “disease” (dilation and reduction in LVEF), and elevated biomarkers was frequent among patients with both moderate and severe AR. These findings are important and highlight how frequently physiological/pathological cardiac adaptation or “damage” coexists with moderate or severe AR and potentially how variable and unpredictable the impact of different LV pressure and volume overload is tolerated by different patients. The observations of cardiac damage in this study are directionally consistent with those reported in prior works and, in terms of LV and LA damage specifically, the modeled scenarios in this study illustrate how accumulation of damage to both of these chambers could confer worse outcomes for these patients.^15^^,^^16^ Whether those structural and functional cardiac alterations are a direct consequence of AR or rather an epiphenomenon due to other coincidental diseases (ie, coronary artery disease, hypertension, long-term atrial fibrillation) is clinically challenging to decipher. Independently, whether the LV or LA injuries are a sequential consequence of a single disease (AR), or instead the result of multiple insults due to several and distinct comorbid diseases, mortality significantly increases with the presence of those factors and most likely still results in a compounded effect of multiple and sustained cardiac insults through time. Whether treating one of those factors, such as the AR, will result in improved prognosis or resolution of cardiac damage remains to be seen prospectively.^15^^,^^17^

In our cohort, patients with a more severe degree of AR were younger, which is different from other valvular diseases. Indeed, patients with aortic stenosis or mitral regurgitation are usually older, and mortality increases with advancing age.^18^^,^^19^ AR etiologies often include bicuspid valve, aortic root, and connective tissue disease, with clinical manifestation typically occurring at a younger age. That said, in spite of the younger age of our cohort, heart failure-related hospitalization was still frequent (∼12%-20%) both for moderate and severe AR. This illustrates the detrimental impact of moderate and severe AR and potentially the need for better therapy for those patients to reduce the economic burden associated with significant AR.

### Study Limitations

The present study has a number of potential limitations that should be acknowledged. First, we used an NLP-based algorithm to extract data from echocardiographic reports provided by each site. Echocardiograms were not reassessed or reinterpreted by an independent core laboratory, and real-world variability in echocardiographic assessment and documentation, perhaps even especially relevant in the context of AR, may be reflected in some aspects of the present work. Second, our population consisted of patients undergoing echocardiographic assessment during an office or hospital visit where AR might not have been the principal issue. Third, it is possible that mortality and treatment data as extracted from each institution’s medical records (ie, as documented by each site) could be incomplete. However, even with this possibility, the present work still illustrates substantial untreated mortality risk for patients with AR, even if the mortality observed might not be fully explained by the AR itself. Fourth, aside from relevant valve-related procedures, data on other therapeutics that may influence cardiovascular (including left ventricular) function were not collected for purposes of the present study. Fifth, these data originate from a subset of the many health systems that exist in the United States, and results from different institutions and regions may differ. Finally, symptomatic status was not assessed in this study. Despite these limitations, this study reflects real-world practice, provides meaningful information regarding challenges in applying guidelines in the community, and identifies potential areas for diagnostic and therapeutic improvement.

## Conclusions

In patients with untreated AR, LV remodeling, LA remodeling, and other markers of cardiac damage are associated with substantial mortality risk both for severe and moderate AR. Further study is needed to determine whether AVR is warranted in patients with less-than-severe AR with at-risk factors.

Perspectives **COMPETENCY IN PATIENT CARE AND PROCEDURAL SKILLS:** The present study indicates that among patients with AR, there may be some cohorts of patients diagnosed with less-than-severe AR with key risk factors who may warrant more careful consideration.

**TRANSLATIONAL OUTLOOK:** Prospective studies will provide more insight into whether key factor-driven assessment and potentially earlier treatment can improve outcomes in patients with AR.

## Funding support and author disclosures

Funding for this work was provided by 10.13039/100019998JenaValve Technology, Inc. Analytical and editorial support was provided by egnite, Inc. Dr Généreux has served as a consultant for 4C Medical, Abbott Vascular, Abiomed, BioTrace Medical, Boston Scientific, Caranx Medical, Cardiovascular Systems Inc, Edwards Lifesciences, GE Healthcare, iRhythm Technologies, Medtronic, Opsens, Pi-Cardia, Puzzle Medical, Saranas, Shockwave, Soundbite Medical Inc, egnite, Inc, and Teleflex; is an advisor to Abbott Vascular, Abiomed, BioTrace Medical, Edwards Lifesciences, egnite, Inc, and Medtronic; has received speaker fees from Abbott Vascular, Abiomed, BioTrace Medical, Medtronic, Shockwave, and Siemens; is a principal investigator of 4C Medical for the AltaValve feasibility study, Cardiovascular Systems Inc for the Eclipse Trial, and Edwards Lifesciences for the EARLY-TAVR and PROGRESS trials; holds equity in Pi-Cardia, Puzzle Medical, Saranas, and Soundbite Medical Inc; and is a proctor for and has received institutional grants from 10.13039/100006520Edwards Lifesciences. Dr Amoroso has served as a consultant and proctor for JenaValve Technology, Inc and Abbott; has received speaker fees from Boston Scientific; has served as a consultant to Edwards Lifesciences and Siemens; is on the advisory board and has an equity interest in Nininger Medical. Dr Thourani is on the advisory board or in research for Edwards Lifesciences, Artivion, Abbott Vascular, AtriCure, JenaValve Technology, Inc, Shockwave, Boston Scientific, Medtronic, and Dasi Simulations; is on the advisory board and is a consultant for egnite, Inc. Dr Rodriguez has received consulting fees, speaker fees, and honoraria from Edwards Lifesciences; consulting fees from egnite, Inc; consulting fees, speaker fees, clinical educator fees, and honoraria from AtriCure; consulting fees, speaker fees, honoraria, and a research grant from Abbott; speaker fees from Phillips; and consulting fees from CardioMech and Teleflex. Dr Sharma has received consulting fees, speaker fees, and honoraria from Edwards Lifesciences, has received consulting fees and has equity interest in egnite, Inc; and has received speaker fees and honoraria from Boston Scientific and Abbott. Dr Pinto has served as a consultant for Abbott Vascular, Abiomed, Boston Scientific, Inari, Philips, Terumo, Teleflex, Biotronik, Medtronic, NuPulseCV, and Magenta; is an employee of and has an equity interest in JenaValve Technology, Inc. Dr Kwon has an equity interest in and is an employee of egnite, Inc. Dr Dobbles has an equity interest in and is an employee of egnite, Inc. Dr Pellikka has received research support from Ultromics and 10.13039/100006520Edwards Lifesciences. Dr Gillam has served as a consultant for Edwards Lifesciences, Medtronic, and Philips; is on the advisory board for egnite, Inc; and has core lab contracts with Abbott, Edwards Lifesciences, and Medtronic for which she receives no direct compensation.
