# Supplementary material for: Mortality Burden for Patients With Untreated Aortic Regurgitation
Source: JACC Adv. 2024 Sep 6;3(10):101228. doi: 10.1016/j.jacadv.2024.101228 (PMC11408366; doi:10.1016/j.jacadv.2024.101228)
Supplement: Supplementary material [file mmc1.pdf]

Supplemental Material

Mortality Burden for Patients With Untreated Aortic Regurgitation

Philippe Généreux, MD; Nicholas S. Amoroso, MD; Vinod H. Thourani, MD; Evelio Rodriguez, MD; Rahul P. Sharma, MBBS; Duane S. Pinto, MD; Michelle Kwon, PhD; Michael Dobbles, MSc; Patricia A. Pellikka, MD; Linda D. Gillam, MD, MPH

Supplemental Table 1. Complete Multivariable Model Output.

Covariate	Hazard ratio (95% CI)
Age	1.04 (1.03-1.04)
Patient sex (male)	1.18 (1.12-1.24)
COPD on O_2	2.17 (1.98-2.37)
Liver disease	1.56 (1.45-1.69)
Moderate or greater MR	1.08 (1.01-1.15)
Moderate or greater TR	1.41 (1.32-1.50)
Mild-to-moderate AR	1.16 (1.09-1.24)
Moderate AR	1.26 (1.18-1.35)
Moderate-to-severe AR	1.35 (1.14-1.60)
Severe AR	2.37 (1.96-2.87)
**LV dilation (LVESDi >25 mm/m^2 and/or LVESVi ≥45 mL/m^2)**	**1.34 (1.21-1.48)**
**LVEF ≤55%**	**1.09 (1.02-1.15)**
**LA dilation (LAVi >34 mL/m^2)**	**1.09 (1.03-1.16)**
**AF**	**1.11 (1.04-1.17)**
**High BNP/NT-proBNP (BNP ≥400 pg/mL and/or NT-proBNP ≥1500 pg/mL)**	**1.71 (1.60-1.84)**
HF-related hospitalization in past year (rolling monthly basis)	3.35 (3.13-3.59)
History of MI (prior to first AR diagnosis)	1.29 (1.19-1.39)
New-onset MI (relative to first AR diagnosis)	1.58 (1.42-1.75)
LV hypertrophy (LV mass index >95 g/m^2 for female / >115 g/m^2 for male)	1.56 (1.30-1.87)
Severe pulmonary arterial hypertension (PASP ≥60 mm Hg)	1.46 (1.12-1.91)
RV dysfunction (by text diagnosis and/or TAPSE <1.6 cm)	1.33 (1.23-1.43)

Bolding indicates covariates considered as key factors of interest in the present study.
AF = atrial fibrillation; AR = aortic regurgitation; BNP = B-type natriuretic peptide; CI = confidence interval; COPD = chronic obstructive pulmonary disease; HF = heart failure; LA = left atrial; LAVi = left atrial volume index; LV = left ventricular; LVEF = left ventricular ejection fraction; LVESDi = left ventricular end-systolic dimension index; LVESVi = left ventricular end-systolic volume index; MI = myocardial infarction; MR = mitral regurgitation; NT-proBNP = N-terminal pro-B-type natriuretic peptide; PASP = pulmonary arterial systolic pressure; RV = right ventricular; TR = tricuspid regurgitation.

Supplemental Figure 1. Main Study Flow Chart.

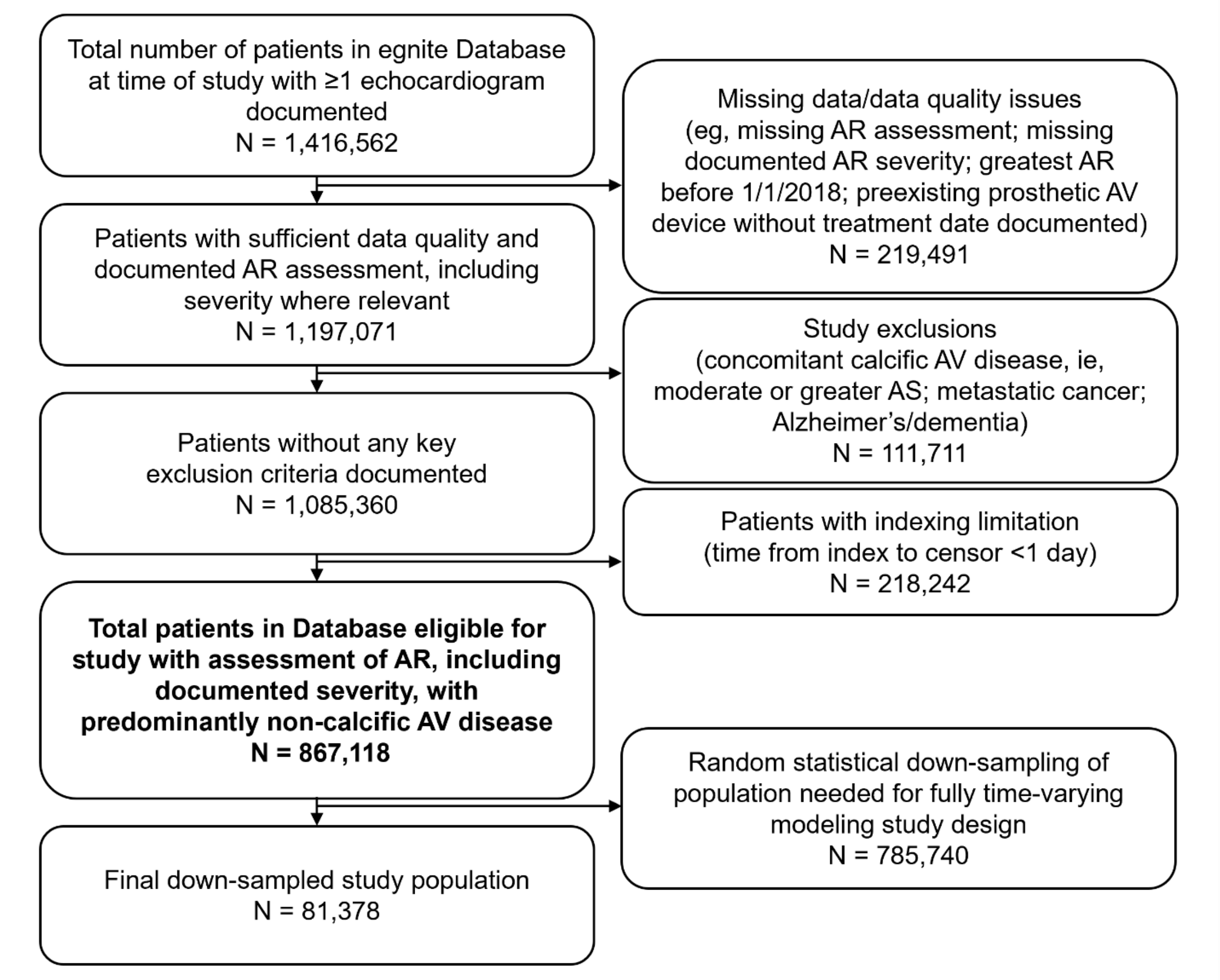

Statistically random down-sampling applied to total eligible population such that no more than 20,000 patients accumulated per cohort when stratified by greatest AR severity.
AR = aortic regurgitation; AS = aortic stenosis; AV = aortic valve.
